# Implementation and Evaluation of the RECAP Framework: A Quality Improvement Initiative

**DOI:** 10.3390/nursrep16020056

**Published:** 2026-02-09

**Authors:** Courtenay R. Bruce, Natalie N. Zuniga-Georgy, Nathan Way, Lenis Sosa, Emmanuel Javaluyas, Terrell L. Williams, Swetha Mulpur, Gail Vozzella

**Affiliations:** 1 System Patient Experience, Houston Methodist System, Houston, TX 77030, USA; nzuniga@houstonmethodist.org (N.N.Z.-G.);; 2 Department of Nursing, Houston Methodist the Woodlands Hospital, The Woodlands, TX 77385, USA; 3 Center for Nursing Research, Education and Practice, Houston Methodist Hospital, Houston, TX 77030, USA; 4 Department of Nursing, Houston Methodist Hospital, Houston, TX 77030, USA; 5 System Analytics, Houston Methodist, Houston, TX 77030, USA

**Keywords:** nursing communication, nursing education, nursing framework, patient care assistant

## Abstract

**Background:** Narration of care (NOC) refers to a nurse’s ability to explain the purposes, goals, and objectives of nursing tasks. In this project, narration of care (NOC) refers to real-time verbal explanation of nursing tasks and should not be confused with the Nursing Outcomes Classification, which uses the same acronym. Although NOC is recognized as a critical skill, little research exists on how to teach it or evaluate its use. A companion article describes the development of a NOC framework. This article focuses on implementation and observed changes during rollout. **Objective:** We aimed to describe the implementation of a discussion-based course designed to teach nurses and patient care assistants (PCAs)—collectively referred to as nursing staff—how to effectively narrate care, and to assess changes observed during the implementation period. **Methods:** We used a mixed-methods, pre- and post-implementation design across seven hospitals over six months (February–August 2023). Quantitative data included pre–post comparisons of Hospital Consumer Assessment of Healthcare Providers and Systems (HCAHPS) scores (baseline: 2022; follow-up: 2024) and structured observations of nurse–patient interactions. Qualitative data from free-text course evaluations were thematically analyzed to contextualize quantitative findings. Integration occurred by comparing themes with observed practice gaps and patient experience trends. **Results: Course Evaluations:** In total, 7341 staff completed the course; 4185 evaluations were submitted. Ninety-five percent reported increased knowledge and rated the course highly. Common strategies cited included teach-back, reducing anxiety through NOC, active listening, and building personal connections. **HCAHPS Comparisons:** Five domains improved significantly post-implementation: care transitions (4.6, *p* = 0.001), cleanliness (3.9, *p* = 0.024), communication about medications (2.3, *p* = 0.042), discharge communication (2.7, *p* = 0.002), and restfulness (2.5, *p* = 0.015). **Practice Observations:** In total, 1281 observations were conducted. Observations indicated frequent use of several NOC-aligned behaviors and opportunities to improve narration of the environment and resolution of patient concerns. **Conclusions:** Improvements in patient experience measures and observed practices coincided with the course rollout. However, given the pre–post, uncontrolled design, causality cannot be inferred.

## 1. Introduction

It is well understood that the nurse–patient relationship is fundamental to high-quality nursing care [1]. In addition to clinical competence, nurses must exhibit strong verbal and non-verbal communication skills [2]. While communication has been widely studied, most research addresses general relational behaviors rather than the specific verbal strategies nurses use during routine tasks. Existing frameworks for patient-centered communication emphasize empathy and shared decision-making [2,3], but they provide limited guidance on what nurses should say in real time to reduce uncertainty and foster trust.

Narration of care (NOC) refers to a nurse’s ability to explain the purposes, goals, and objectives of nursing tasks as they occur. Unlike broader communication constructs, NOC focuses on real-time narration of procedures and environment to enhance patient understanding and comfort. This definition of NOC should not be confused with the Nursing Outcomes Classification, an outcome measurement system that shares the same acronym but differs entirely in purpose and scope. Although NOC is recognized as important, few studies have examined structured approaches to teaching it or evaluating its integration into practice. A companion article describes the development of a NOC framework [4]. This article examines its implementation and observed changes during rollout.

We implemented a discussion-based course designed to teach nurses and patient care assistants (PCAs)—collectively referred to as nursing staff—how to effectively narrate care. Outcomes were assessed through

- **Course evaluations** (staff feedback and self-reported learning);
- **Hospital Consumer Assessment of Healthcare Providers and Systems (HCAHPS) scores** (pre–post comparisons across seven hospitals);
- **Practice observations** (to assess NOC behaviors and identify improvement opportunities).

Our evaluation was guided by Kirkpatrick’s framework [5]: Level 1 (learner reactions) and Level 2 (knowledge gains) were assessed through course evaluations; Level 3 (behavioral change) through structured observations; and Level 4 (organizational outcomes) through HCAHPS comparisons. While communication training programs often focus on knowledge gains alone [6], few link training to organizational outcomes, and those that do rarely address narration of care specifically.

HCAHPS was selected because it is a widely used patient-experience measure in U.S. hospitals. Domains related to communication and care transitions align with NOC principles, while cleanliness and restfulness may reflect perceived attentiveness and environmental narration. However, HCAHPS has limitations: scores are aggregated at the hospital level, influenced by multiple initiatives, and may not isolate the effect of a single intervention. These considerations informed our interpretation of findings.

Table 1 summarizes the NOC framework and course content, which are essential for understanding project outcomes. The framework consists of five principles represented by the acronym RECAP: reduce uncertainty, explain the environment, maintain calm, assume nothing, and personalize connections.

## 2. Methods

### 2.1. Study Design

We used a mixed-methods, pre–post implementation design to evaluate changes associated with a discussion-based course on narration of care (NOC). Quantitative data included Hospital Consumer Assessment of Healthcare Providers and Systems (HCAHPS) (Baltimore, MD, USA) scores and structured observations of nurse–patient interactions [7]. Qualitative data included free-text responses from course evaluations. Data were collected in parallel and integrated during interpretation to contextualize quantitative trends.

The primary outcome was a change in HCAHPS communication domain scores. Secondary outcomes included course evaluations (learner reactions and self-reported knowledge and skill gains) and observed NOC behaviors. We hypothesized that course implementation would coincide with improved patient experience scores and increased use of NOC behaviors.

The course was rolled out across seven hospitals over six months (February–August 2023). Pre-intervention baseline (calendar year 2022) and post-intervention follow-up (calendar year 2024) were selected to reduce seasonal variation and allow time for practice uptake, though this introduces potential confounding by secular trends and concurrent initiatives. The design did not include a control group or phased rollout. Findings should be interpreted in this context. Given the quality-improvement nature of the project, no formal sample size calculation or power analysis was performed, and analyses were exploratory.

The hospital review board determined this project would not be regulated as human subject research. All data were aggregated and de-identified prior to analysis.

### 2.2. Population and Setting

This project took place within a quaternary medical system in Houston, Texas, comprising one academic medical center (>1386 beds) and six community hospitals (200–500 beds each). The turnover rate for this hospital system is 12.7% for medical–surgical nurses, with two hospitals experiencing 2% and 3% lower rates of turnover, respectively, compared to the other five hospitals. All hospitals have a 5:1 patient-to-nurse ratio for medical-surgical units. There were no significant changes in nursing workflows or staffing ratios during this project period.

The intervention targeted nurses and patient care assistants (PCAs) working on medical–surgical inpatient units. Exclusion criteria included outpatient nursing staff, nurse executives, critical care nurses, and research administrative staff who do not provide direct bedside care. Across the seven hospitals, patient and nurse demographics were broadly similar, and baseline HCAHPS performance was comparable system-wide. Contextual factors during the project period included concurrent patient experience and nursing initiatives, which may have influenced feasibility and outcomes.

Although HCAHPS is aggregated at the hospital level, it predominantly reflects experiences on medical–surgical units, which were the primary focus of our project. Only a small proportion of responses come from mother–baby units and Intensive Care Units (ICUs), which were not part of the intervention, so we expect minimal attribution concerns related to unit-level mismatch.

### 2.3. Instruments

The course evaluation survey was locally developed by nurse education administrators and not psychometrically validated, as is typical in formative quality improvement projects. The results should be interpreted as descriptive feedback. Two questions assessed perceived improvement in knowledge and skill:

- “How much has your knowledge on narration of care increased as a result of this offering?”
- “How much has your skill of narrating care increased as a result of this offering?”

Responses used a 5-point Likert scale (ranging from not increased to greatly increased). These questions are part of the standardized evaluation form required by the continuing nursing education program, which limits the extent to which wording can be modified. Additional questions evaluated course organization, audiovisual quality, and scheduling (5-point scale: poor to excellent), likelihood of recommending the course (1–10 scale), and overall satisfaction (1–10 scale).

One free-text field asked learners to identify a new learning and describe its potential impact on performance or patient outcomes. As self-reported measures were collected exclusively post-course, results may be subject to attribution bias.

Patient experience was measured using the HCAHPS survey, a 29-question, publicly reported, government-mandated instrument [7]. Domains include nursing communication, responsiveness, care transitions, and others (see Table 2), and we evaluate impacts using aggregated scores. HCAHPS scores were analyzed using two exploratory approaches that utilize different units of analysis. In the first approach, we calculated each hospital’s pre-intervention (2022) and post-intervention (2024) scores by averaging its values across all 10 HCAHPS domains. Because these averages came from 10 domain level scores, the sample size for this analysis was *n* = 10. The Mann–Whitney test was then used to compare the aggregated pre- and post- implementation scores. In the second approach, we calculated each domain’s pre- and post-intervention scores by averaging the corresponding values across all seven hospitals. Because these domain level averages were based on 7 hospital specific scores, the sample size for this analysis was *n* = 7. Mann–Whitney tests were used again for pre- and post- implementation comparisons. Because the sample sizes are small (*N* = 10, *N* = 7), both analyses were considered exploratory, and inferential tests should be interpreted descriptively.

Practice observation was assessed using an internally developed tool created by the System Patient Experience Team (see Table 3). The tool defines the RECAP principles and uses a 5-point Likert scale to rate observed behaviors, with a free-text item for improvement suggestions. Although observers met before data collection to align scoring expectations and ensure consistency, the tool lacks a standardized rubric or anchor examples, and no formal reliability testing was conducted. We noted very little variation in numeric ratings during implementation. However, given these limitations, we elected to report only general themes from observations rather than quantitative scores.

### 2.4. Procedures

Course evaluation surveys were completed via QR code at the end of each class and aggregated in Microsoft Excel. Because completion of the evaluation is required for learners to receive continuing education credit, most participants complete the survey regardless of satisfaction level, reducing the likelihood of selection bias typically associated with voluntary end-of-class evaluations.

Thirty-five observers were trained through a 2 h virtual and in-person train-the-trainer session led by the lead author. Training covered what to look for during clinical observations, how to provide supportive feedback on narration skills to nursing staff, the number of observations per unit, and how to use the evaluation template (Table 3). The observation tool was digitized into a rounding app, accessible on tablets or phones during clinical encounters.

Observations were conducted randomly based on which nurses or PCAs were actively engaged in patient interactions at the time when observers approached the unit. Of all staff approached, none refused participation, and all planned observations were completed. Observers were blinded to prior performance when conducting observations, and most were unaware of unit-level HCAHPS scores. After observing 2–3 patient interactions, observers provided one-on-one verbal feedback to nursing staff outside patient rooms, typically in a quiet corner of the unit to maintain privacy.

Because observers also acted as coaches, we acknowledge the potential for observer bias and reactivity (Hawthorne effect) [8,9], as staff may have temporarily enhanced narration behaviors when being observed. These limitations were considered when interpreting findings.

### 2.5. Data Analysis

For HCAHPS, we compared scores from before and after NOC course implementation. The pre-implementation period was January-December 2022, and the post-implementation period was January–December 2024. These timeframes were chosen to capture a full year of data before and after implementation. Since most of 2023 was dedicated to this system-wide rollout, 2022 and 2024 served as clean pre- and post-timeframes.

HCAHPS data were analyzed using Mann–Whitney U tests with a significance threshold of *p* < 0.05 in R (version 4.4.1), given the small sample size (seven hospitals) and non-normal distribution of HCAHPS score proportions.

Free-text fields were analyzed using thematic analysis. Two individuals with similar professional backgrounds in patient experience—both trained in qualitative review and working within the same system—conducted the coding. Because the coders shared comparable training and roles, reflexivity concerns were minimized [10], though we acknowledge that their professional orientation toward patient experience may have influenced interpretation. Both coders independently reviewed all comments, marked salient text, and developed an inductively derived codebook consisting of major categories and subthemes. The coders used anchor quotations to refine code definitions and ensure consistent application.

Discrepancies in coding were resolved through in-person meetings in which coders discussed divergent interpretations and reached consensus on the most appropriate code. In the rare instance (one situation) when consensus was not immediately clear, a third author was consulted to adjudicate [11]. Data saturation was not a central concern given the large volume of comments. Instead, the goal was to capture the breadth of perspectives represented across all responses [12]. Interrater reliability for initial coding was calculated using percent agreement (88%), which provides descriptive insight into coder alignment but should be interpreted cautiously, given the absence of Cohen’s kappa.

## 3. Results

Of 8603 eligible staff (7005 eligible RNs and 1598 eligible PCAs), 5478 RNs and 1288 PCAs attended the course (85% participation), with attendance varying across hospitals (range: 77–92%). Because completing the evaluation was required for continuing education credit, evaluation response rates were higher for nursing (*n* = 3937/5478; 72%). On the other hand, PCAs were not required to complete the evaluation for continuing education credit, and therefore only 186 PCAs (14%) completed the course evaluation.

Most learners reported increased knowledge and skills related to narration of care. Specifically, 2971 (71%) indicated their knowledge “greatly increased,” 700 (17%) “fairly increased,” and 309 (7%) “somewhat increased,” with the remainder selecting minimal or no increase. On teaching effectiveness, 3146 (75%) rated the instruction as “extremely well taught,” 783 (19%) as “very well taught,” and 232 (6%) as “well taught.”

Regarding course organization and content, 75% rated the course “excellent” and 18% “good,” with similar ratings for schedule, format, and audiovisual quality. The likelihood of recommending the course was high, with 91% rating it 7–10 on a 10-point scale.

A total of 4188 comments were submitted. Themes reflected both reinforcements of course content and participants’ reflections on how NOC concepts intersect with their existing communication habits, professional identity, and unit culture (Table 4). Five overarching themes emerged, though subtheme variation was noted across roles and settings. Interrater reliability for initial coding was 88% (percent agreement).

Theme 1: Eliciting and Assessing Patient Understanding

This was the most frequently referenced concept, appearing in 1945 of 4188 comments (46%). While many responses aligned with course content (e.g., teach-back), others referenced role-specific challenges, particularly among PCAs who highlighted navigating multilingual communication barriers and time constraints. Here is an illustrative quote: “*By leveraging interpretative services for non-English speaking patients, I can significantly impact performance improvement by enhancing communication… and patient empowerment*.”

Theme 2: Reducing Uncertainty and Patient Anxiety

Comments related to reducing patient uncertainty or anxiety appeared in 364 of 4188 comments (9%). Nurses frequently described how pace of care, task load, or unit workflow affected their ability to reassure patients. Some reflected on how NOC could help bridge mismatches between patient expectations and clinical realities. As explained by a nurse: “*I’m always trying to move quickly. I learned that spending a few more minutes explaining the plan for the day, and asking for [the patient’s input] can help ease their anxiety.*”

Theme 3: Presence, Listening, and Attentiveness

This theme appeared in 148 comments (4%), with greater representation among nurses newer to the system and PCAs who described balancing task-oriented responsibilities with relational communication. As one nurse wrote: “*The course reminded me to slow down and turn attention toward the family, too.*”

Theme 4: Building Personal Connections

Personal connection was noted in 120 comments (3%) and often reflected individual communication styles, cultural background, or unit culture. As one nurse explained, “*When I take the time to explain to patients what, when, and how things are being done, this can build rapport and connection.*”

Theme 5: Perceived Lack of Relevance for Experienced Nurses

There were 43 comments (1%) suggesting that experienced nurses felt the course was most relevant for newer, less experienced clinicians. These comments often cited the fact they felt they regularly employed the strategies we outlined in the course, such as, “*This course was more of a review of common practices that I already use,*” or “*I already incorporate everything learned in this class in my daily practice.*” Of note, even these comments that suggested non-relevance often expressed appreciation for a refresher course or examples they had not previously entertained, as explained by this nurse: “*Most of the course was reiterating education I already have, but one thing I was reminded about was to make sure I am providing a rationale and always ask for permission before beginning a task that required touching or movement.*”

HCAHPS Comparisons. HCAHPS domain sample sizes increased from 2022 to 2024 across all measures. In 2022, sample sizes ranged from about 13,800 to 23,300, depending on the month. By 2024, they had grown to roughly 20,100 to 29,400. Despite these increases, overall survey response rates and patient demographic characteristics remained stable between the two periods, indicating no meaningful changes in patient mix.

Five domains improved significantly post-implementation: care transitions (4.6, *p* = 0.001), cleanliness (3.9, *p* = 0.024), communication about medications (2.3, *p* = 0.042), discharge communication (2.7, *p =* 0.002), and restfulness (2.5, *p* = 0.015) (see Table 5). We can see a wide confidence interval for the tests conducted by hospital (*N* = 10 domains), showing there may be factors that may need to be considered. However, the tests by domain (*N* = 7 hospitals) show a narrower confidence interval, as well as significant *p*-values (Table 5). In addition to the five domains that improved significantly post-implementation, we examined changes in the nurse communication domain, which is conceptually most aligned with NOC. Nurse communication scores increased by 0.99 points post-intervention; however, this change was not statistically significant. Less than 1% of responses came from mother–baby units and ICUs, which were not part of the intervention. Therefore, there were minimal attribution concerns related to unit-level mismatch.

Practice Observation: In total, 1281 observations were conducted, meaning 1281 nurses or PCAs were evaluated by 35 trained observers in nurse–patient interactions. This represented 26% of the hospital system’s nursing staff workforce during the project period, though some individuals (fewer than 10) may have been evaluated more than once.

Observers frequently noted use of NOC-aligned behaviors, particularly related to reducing uncertainty (R), maintaining a calm and sincere demeanor (C), and developing personal connections (P). Interrater reliability on the free-text comments was 90%. Opportunities for improvement focused on nuances of RECAP and missed opportunities (see Table 6). For example, while often positive, observers noted that task-driven behavior sometimes limited meaningful engagement, especially with highly talkative patients (personal connection or the P in RECAP). Routine IV-related tasks (e.g., flushes, occluded lines, medication changes) were occasionally performed without explanation, assuming patient familiarity, which is the principle of assume nothing (A).

## 4. Discussion

There is consensus that clinicians’ communication skills can be improved with the right training and right teachers, owing to a large body of available descriptive research [13,14,15,16]. However, little research has been done on course implementation and outcomes beyond the use of simulated patients [14,17,18]. Most courses evaluating nursing communication skills have focused exclusively on standardized patients or simulation methodologies, like role play or the use of manikins. Outcome assessments focus on knowledge gains. Few communication skills courses have evaluated outcomes from patients’ perspectives or examined organizational-level metrics. Further confounding matters, little association has been found between the number or quality of the courses nurses take and their own confidence and competence in nursing–patient communication [19,20].

We sought to address this gap using a combination of objective and subjective measurements. By adding a patient-centered objective dimension of HCAHPS (one that was not dependent on self-assessments or faculty-designed measurement instruments), we could have confidence that the course had some positive impacts on patients, not just nurses [21]. This is one way our work is innovative and contributes to the existing literature. Moreover, by adding an observation component—observing whether and how NOC practices were incorporated into nurse–patient clinical encounters in actual practice—we incorporated an additional dimension that explored whether and how practice was changed because of the program, adding a level of practicality and sustainability not previously examined.

## 5. Interpretation of Results

The most salient finding was that patient-centered outcomes and organizational metrics showed generally positive trends over the course of implementation. However, because the project design does not allow the course to be isolated as the causal factor, these patterns should be interpreted as associations rather than evidence of effectiveness.

With that in mind, several of the observed improvements aligned with domains that were emphasized in the course: communication about medications, discharge education, and care transitions. Course case studies focused on explaining medication purposes and side effects, offering choices in medication routines, narrating the discharge process, and clarifying steps involved in transitions between care settings. These elements parallel the areas in which HCAHPS scores improved, though we cannot determine whether the course directly contributed to these changes.

In contrast, improvements in the restfulness and cleanliness domains were not anticipated. We offered possible explanations based on course content, such as setting expectations for nighttime interruptions or explaining clutter removal, but it is important to note that we did not measure whether participants implemented these practices, nor did we collect qualitative data on patient perceptions in these areas. As a result, any linkage between course content and improvements in these domains should be considered hypothesis-generating.

Nurse communication scores also warrant a nuanced interpretation. While nurse communication is indeed a complex, multidimensional concept that may not be fully addressed by a single training, several additional factors may have contributed. First, HCAHPS nurse communication items may not be tightly aligned with the specific behaviors targeted in the course. Second, many units began the project period with already-high baseline scores, raising the possibility of ceiling effects that limited room for detectable improvement [22,23,24]. Finally, concurrent organizational initiatives or shifts in patient acuity period may have influenced scores in ways unrelated to the course.

Another key finding is that the course produced inconsistent changes in observable behavior, with no clear pattern across units or hospitals. This aligns with the prior literature indicating that increased knowledge does not reliably translate into behavioral change in clinical settings. Although nurses reported intentions to apply responsiveness strategies, such as unpacking patient concerns or explaining the steps they would take to address an issue, observers still noted instances in which patient concerns (e.g., pain) elicited passive responses that lacked explanation or follow-through. This discrepancy suggests that even when nurses value and understand these practices, contextual barriers such as competing priorities, workload strain, clinical demands, or cultural norms within units may constrain their use [24]. Future work should examine these contextual factors systematically to better understand what supports or inhibits sustained adoption.

### Comparison with the Literature

Nursing staff reported knowledge gains after course delivery, consistent with previous research on nurse–patient communication training [25,26,27]. Participants also expressed high satisfaction with the course and intended to integrate the strategies into practice, aligning with findings from other communication skills interventions [28]. However, unlike many prior studies, we were able to observe changes in practice during the implementation period. Importantly, we do not claim that this approach is unique within the full landscape of communication improvement efforts. Rather, our contribution lies in demonstrating how structured observation and coaching can function within a large health system.

A key element of the implementation was the use of trained observers who also served as communication coaches. While this approach offers advantages—such as enabling real-time, behavior-specific feedback and providing a consistent framework for identifying effective communication behaviors—it also carries methodological trade-offs. The dual role of observer–coach may introduce potential observer bias or loss of independence, and the presence of observers may contribute to Hawthorne effects, in which staff temporarily modify their behavior because they are being watched [8,9]. These limitations should be considered when interpreting the behavioral data, and future research may benefit from separating the observation and coaching functions.

Through observational data, we found that some strategies nurses intended to use were not fully realized in practice. While the existing literature on task-focused care offers one plausible explanatory framework [29,30,31,32], interpretation should be situated in the context of this health system. Multiple factors—such as workload pressures and competing demands—may have influenced the degree to which nurses were able to apply the strategies consistently. Anecdotal comments from observers and unit leaders suggest that nurses often had limited time for in-depth communication during peak activity periods. Understanding how workload and unit-level priorities interact with communication behaviors will be an important direction for future inquiry.

## 6. Limitations

The pre–post, uncontrolled quality improvement design limits causal inference, and the use of 2022 and 2024 as comparison years introduces potential confounding from concurrent initiatives and broader system-level changes. HCAHPS scores are aggregated at the hospital level, which includes units outside the intervention scope and may dilute measurable effects from medical–surgical units. It is not possible to make an assertive claim that the NOC course was solely responsible for improvements in HCAHPS, given that this project was a pre–post-implementation comparison and not a randomized trial [33]. A randomized, controlled trial might have been the purest way of assessing the impacts of a singular intervention [34], but such a method is not operationally feasible in quality improvement projects such as this where all nurses receive training. The lack of a control group and random sampling weakens generalizability.

A second limitation is the lack of systematic analysis of sustainment practices. While we can anecdotally report that HCAHPS outcomes after one year of intervention are similar to those at six months, we cannot confirm that the course caused this sustainment or identify the factors contributing to it.

The course evaluation data were based on self-report measures collected post-training only and relied on a standardized continuing education survey format, which restricts item modification. Third, the observation tool used to assess practice observation was internally developed without formal validation or standardized scoring anchors. Although observers met to align scoring expectations and demonstrated minimal variation during implementation, numeric ratings were not subjected to interrater reliability testing and are therefore not reported. Additionally, observers served both evaluative and coaching roles, creating the potential for observer bias, and staff awareness of being observed may have temporarily enhanced NOC behaviors (Hawthorne effect). The qualitative analysis, while conducted by coders with similar training and backgrounds that helped reduce reflexivity concerns [10] relied on percent agreement (88%) and did not explicitly aim for data saturation given the large volume of comments.

A further limitation relates to the composition of the evaluation and observation samples. The course evaluations were completed primarily by registered nurses, and the majority of practice observations were also conducted with nurses. Unfortunately, observers did not record whether they were observing nurses or PCAs. Because RNs and PCAs have distinct scopes of practice and role expectations, this imbalance limits direct comparability between self-reported outcomes and observed behaviors. Many of the findings, particularly those related to clinical decision-making, communication of care plans, and integration of narrative opportunities into nursing workflow are therefore most applicable to RNs. We chose not to exclude PCAs from the analysis because, although representing a smaller dataset that limits generalizability, they engaged in similar forms of environmental and situational narration, and their course evaluation feedback reflected intentions to apply many of the same strategies as nurses. Nonetheless, the role variation between groups should be considered when interpreting the findings.

These limitations should be considered when interpreting the findings, which are intended to inform ongoing system quality improvement.

### Strengths

This project also has several notable strengths. It was implemented across a large, diverse, multi-hospital system, enhancing the validity and practical relevance of findings. The evaluation design incorporated multiple data sources, including learner feedback, structured observations, and hospital-level patient experience metrics, providing a broad, multi-level understanding of how narration-of-care practices were received and enacted in real clinical environments. The use of Kirkpatrick’s framework [5] enabled an organized assessment across reaction, learning, behavior, and organizational levels, which is uncommon in studies of nursing communication training.

Additionally, the inclusion of direct practice observations offered insight into real-time bedside communication behaviors, an area often overlooked in nursing education research. The qualitative analysis was conducted by coders with similar professional training, reducing interpretive variability, and the large number of course participants and observations strengthens confidence in the representativeness of the findings. Finally, the project was fully integrated within routine clinical operations, demonstrating feasibility and sustainability for large-scale quality-improvement efforts aimed at enhancing patient-centered communication.

## 7. Conclusions

The findings from this quality improvement project demonstrate positive associations between the implementation period of the NOC course and several patient-centered and organizational outcomes. The qualitative and quantitative data provide insights into how NOC-aligned behaviors may function in practice, as well as where implementation challenges persist.

Several HCAHPS domains improved during the project period. They highlight areas in which communication-focused interventions may hold promise and inform priorities for ongoing practice development. For example, recurring gaps identified through observations, particularly around escalation of pain and comfort needs, logically guide the content of our next steps. In response, we plan to develop targeted “refresh” training and structured huddle-based case discussions that directly address the specific communication challenges nurse leaders continue to observe in real time. These planned activities are therefore informed by, but not justified by, the current findings. They represent iterative adaptations consistent with continuous quality improvement.

Given the limitations of this project, future research is essential. Multi-site or experimental studies will be particularly important for determining whether NOC practices can produce measurable improvements under more rigorous conditions. Additionally, future work should capture unit-level contextual variables such as staffing and workload and examine longitudinal sustainability of communication behaviors. This project represents an initial step in a broader program of inquiry into the NOC approach, providing foundational insights to guide more definitive studies.

## Figures and Tables

**Table 1:** The Narration of Care Framework (RECAP Principles), Examples, and Applications.

RECAP Principle	Definition or Concept	Expected Benefit	Course Example
**R: Remove uncertainty**	Explain what you are doing and why you are doing it to remove uncertainty.	Removing uncertainty reduces patient anxiety and helps prepare patients mentally and physically for any type of treatment or procedure.	40-year-old woman undergoing her first mammogram. There is no historical baseline imaging on file. The mammogram technician explains to the patient that additional imaging is routine practice for patients who have no baseline history available for clinicians. As a result of that conversation, the patient is not alarmed when she is called in for repeat imaging.
**E: Explain the environment**	Explain the clinical environment by describing the purpose of any equipment, sounds, and goals of monitors, readings, and alarms.	Providing explanations for the environment can increase knowledge and reduce anxiety.	76-year-old woman with Alzheimer’s disease having her blood pressure taken. The PCA narrates to the patient that she would need to take her blood pressure “every few hours,” but fails to tell the patient that the cuff would tighten, why that would happen, and when it would release. As a result, the patient becomes alarmed and begins crying when the cuff tightens.
**C: Be calm and sincere**	Convey calmness and sincerity with appropriate tone and mindful body language.	Demonstrating calmness and sincerity reduces the chances of a hurried or task-oriented demeanor, which unintentionally creates the perception that nursing staff are hurried, not listening, and not being present.	33-year-old male being turned by a PCA who fails to explain what “turning” means, why he would need to do it, and what the patient might experience because of it. The PCA fails to confirm the patient is ready for turning, fails to stop when the patient expresses uncertainty, and does not reassess the patient’s comfort.
**A: Assume Nothing**	Treat all patients similarly, regardless of education level, previous hospitalizations, or medical sophistication. Provide patients with the opportunity to ask questions.	Providing clear information in words that patients can understand allows them to know what to expect.	82-year-patient has been on Gabapentin for years, so the nurse does not mention side effects, compared to a different nurse saying to the same patient, “You’ve been on Gabapentin for several years. What side effects do you experience at home?”
**P: Personal connection**	Develop a personal connection with patients by learning what is most important to them.	Mentioning topics that are of interest to the patient show respect, active listening, and intentionality.	30-year-old in a hospital bed, with a handwoven blanket, a book, a children’s illustration on his wall, and a picture of his dog in the background. Course facilitators ask the nursing staff how to build a personal connection using those objects.

**Table 2:** Hospital Consumer Assessment of Healthcare Providers and Systems (or HCAHPS) Survey.

Domain	Questions
**Overall Rating of Hospital**	1. Using any number from 0 to 10, where 0 is the worst hospital possible and 10 is the best hospital possible, what number would you use to rate this hospital during your stay?
**Would Recommend Hospital**	1. Would you recommend this hospital to your friends and family?
**Communication with Nurses**	1. During this hospital stay, how often did nurses treat you with courtesy and respect? 2. During this hospital stay, how often did nurses listen carefully to you? 3. During this hospital stay, how often did nurses explain things in a way you could understand?
**Communication with Doctors**	1. During this hospital stay, how often did doctors treat you with courtesy and respect? 2. During this hospital stay, how often did doctors listen carefully to you? 3. During this hospital stay, how often did doctors explain things in a way you could understand?
**Care Transitions**	1. When I left the hospital, I had a good understanding of the things I was responsible for in managing my health. 2. When I left the hospital, I clearly understood the purpose for taking each of my medications. 3. During this hospital stay, staff took my preferences and those of my family or caregiver into account in deciding what my health care needs would be when I left.
**Communication Re: Medicines**	1. Before giving you any new medicine, how often did hospital staff tell you what the medicine was for? 2. Before giving you any new medicine, how often did hospital staff describe possible side effects in a way you could understand?
**Discharge Information**	1. During this hospital stay, did doctors, nurses or other hospital staff talk with you about whether you would have the help you needed after you left the hospital? 2. During your hospital stay, did you get information in writing about what symptoms or health problems to look out for after you left the hospital?
**Response of Hospital Staff**	1. During this hospital stay, when you asked for help right away, how often did you get help as soon as you needed?
**Cleanliness of Hospital Environment**	1. During this hospital stay, how often were your room and bathroom kept clean?
**Restful Hospital Environment**	1. During this hospital stay, how often was the area around your room quiet at night? 2. During this hospital stay, how often were you able to get the rest you needed? 3. During this hospital stay, did doctors, nurses and other hospital staff help you to rest and recover?

**Table 3:** Narration of Care Observer Auditing Tool.

RECAP Narration of Care Coaching and Mentoring Tool			
Coach: ____________________	Staff Member: ____________	Dept/Unit: ________	Date: _____________
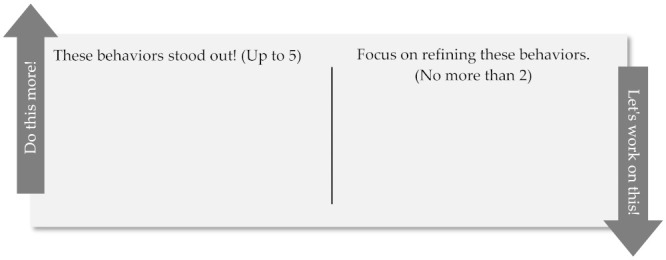			
Standards		Demonstrated Skill Opportunity 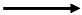 Strength	
Calm and Sincere Before a word is spoken, convey calmness and sincerity with posture, eye contact, and smile.		1 2 3 4 5	
Personal Connection Makes a personal connection by listening intently and making note of something important to your patient (people, places, pets, possessions) to reference later.		1 2 3 4 5	
Explain Environment Present their room/procedural area as a foreign environment by using words to explain what can be seen, heard, or felt.		1 2 3 4 5	
Remove Uncertainty Words can remove uncertainty if you include why the task is necessary and what they will see, hear, feel, expect, and/or smell.		1 2 3 4 5	
Assume Nothing Assume nothing about the patients’ medical literacy or comfort in the environment and ask meaningful questions to close the loop.		1 2 3 4 5	

**Table 4:** Learner Evaluations and Strategies to be Used in Practice.

Strategy That Would Be Used in Practice	Specific Learner Comment
Elicit patient understanding	“I never realized how important non-verbal clues were to suggest [a lack of understanding]. I can explore understanding and seek clarification. Then I can do a teach back and confirm the patient is satisfied with the information.”
	“By leveraging interpretative services for non-English speaking patients, I can significantly impact performance improvement by enhancing communication, ensuring regulatory compliance, and ultimately improving patient outcomes through increased understanding, improved safety, and patient empowerment.”
Reduce uncertainty and mitigate anxiety	“The reality is that sometimes when you are loaded with tasks, you tend to forget the importance of explaining the tasks that might cause confusion and anxiety to the patient. With this class, it reminded me to be more mindful of my actions and to NOC.”
	“I learned the importance of the five principles of care narration using the RECAP model. Implementing this will impact my performance because the patients will be more comfortable, feel that they have a sense of control over their care, and feel valued, giving me the opportunity to grow in my communication as a health professional.”
	“I believe that the RECAP skills are highly effective. I’ve been using narration of care for years, as a former educator, I find that “explaining and teaching as you go” while at the bedside goes a long way in easing patient (and family), anxiety and give them peace of mind in knowing what to expect.”
	“As a registered nurse in L&D, I do my best to narrate care down to glove changes between medication administrations and assessments. These little actions truly make a difference in care and build trust between patients. While it might be regarded as a bit “extra” to peers, we are already taking care—might as well talk through it. I am glad this class has been introduced to our staff. Hospital staff do their jobs routinely and may forget this could be someone’s first hospitalization. As I recently was a patient family member at the bedside, I found myself having to narrate care for my loved one to reassure them they were being taken care of. This should be the gold standard at every hospital.”
Slow down to listen and be fully present	“I always focused on the patient. But [the course] reminded me to slow and turn attention towards the family, too.”
	“Narration of care will greatly increase patient outcomes because it provides patient comfort and understanding. Not only will the patient be more knowledgeable about what you’re doing and why, but it will make them more likely to ask or voice any questions or concerns that they may have because they trust you and know that you don’t mind explaining things for their understanding.”
Build personal connections	“When I take the time to explain to patients in steps on what, when, how certain things are being done, this can build rapport and connection with patients.”
	“I will use objects in the room to initiate conversation about topics that are not healthcare-related to give me insight into what is important to the patient.”
Perceived lack of relevance for experienced nurses	“Explaining the environment is something I already do but some of the advice they have about doing it better will be helpful”
	“I feel that I already do the narration of care as a 13 year nurse. I am all about educating my patients to why I am doing the actions that I am doing in every day care.”
	“I think it was nice to see the information presented like this. I am a nurse who constantly narrates care already but I am glad it is being focused on system wide”
	“I think the content presented here is truly valuable, however not to an experienced nurse who already practices these techniques. I think this presentation is geared more towards newer nurses/hospital employees”

**Table 5:** Changes in HCAHPS Scores Using Two Analytical Approaches.

By Hospital									
**Location**	***N***	**Average of Pre-Implementation Score January–December 2022**	**Average of Post-Implementation Score January–December 2024**	**Average of Score Difference**	**Mann-Whitney Test for Difference** **(Hypothesis: Pre-Implementation Is Less Than Post-Implementation)**				
					**Difference**	**CI for Difference**	**Achieved Confidence**	***p*-Value**	
Hospital #1	10	73.65	77.07	3.42	−3.15	(−13.2, 5.4)	95.55%	0.154	
Hospital #2	10	75.47	77.08	1.61	−1.3	(−11.8, 9.2)	95.55%	0.367	
Hospital #3	10	72.86	74.52	1.66	−1.5	(−13, 9.6)	95.55%	0.236	
Hospital #4	10	73.95	75.67	1.72	−1.55	(−13.4, 8.2)	95.55%	0.214	
Hospital #5	10	75.12	76.88	1.76	−1.75	(−12.8, 10.8)	95.55%	0.285	
Hospital #6	10	74.7	77.7	3	−2.95	(−15.7, 10.8)	95.55%	0.137	
Hospital #7	10	72.38	74.68	2.3	−1.55	(−11, 8.5)	95.55%	0.26	
**All Hospitals**	**70**	**74.02**	**76.23**	**2.21**	**−2**	**(−4.5, 0.5)**	**95.04%**	**0.055**	
**By Domain**									
**Domain**	***N***	**Average of Pre-Implementation Score January–December 2022**	**Average of Post-Implementation Score January–December 2024**	**Average of Score Difference**	**Mann-Whitney Test for Difference** **(Hypothesis: Pre-Implementation Is Less Than Post-Implementation)**				
					**Difference**	**CI for Difference**	**Achieved Confidence**	***p*-Value**	
Care Transitions	7	56.79	61.23	4.44	−4.6	(−6.4, −2.2)	95.17%	0.001	
Cleanliness of Hospital Environment	7	78.36	81.54	3.19	−3.9	(−6.6, 0.0)	95.17%	0.024	
Communication Re: Meds	7	63.04	65.26	2.21	−2.3	(−5.7, 0.9)	95.17%	0.042	
Communication with Doctors	7	79.97	80.96	0.99	−0.9	(−4.5, 0.5)	95.17%	0.222	
Communication with Nurses	7	81.17	82.73	1.56	−1.7	(−4.5, 0.5)	95.17%	0.101	
Discharge	7	86.39	88.93	2.54	−2.7	(−3.8, −1.4)	95.17%	0.002	
Overall Rating of Hospital	7	80.44	81.99	1.54	−1.4	(−4.5, 0.5)	95.17%	0.153	
Response of Hospital Staff	7	65.97	68.09	2.11	−2	(−7.7, 3.5)	95.17%	0.153	
Restful Hospital Environment	7	65.90	68.64	2.74	−2.5	(−7.1, −0.2)	95.17%	0.015	
Would Recommend Hospital	7	82.16	82.93	0.77	−0.7	(−4.5, 0.5)	95.17%	0.261	

Note: Approach 1—“By Hospital” Analysis (N = 10 domain level scores used to calculate each hospi-tal’s pre/post averages). Approach 2—“By Domain” Analysis (N = 7 hospital level scores used to calculate each domain’s pre/post averages). All tests and results are exploratory due to small sample sizes.

**Table 6:** Observer Feedback.

Opportunities	Clinical Examples	Specific Observer Feedback to Nursing Staff
**Removing Uncertainty**	Setting expectations on sleep and nightly routines (vitals, rounds, medications, lab draws, alarms, environmental noises).	The observer noted that the nurse completed care tasks without outlining what the patient should expect overnight. *“Let’s work on explaining to the patient what to expect overnight, including vital checks, rounds, medication passes, lab draws, and noises in the hospital. Remember to assume the patient has never been to the hospital.”*
	Clarifying what will happen next during care activities (e.g., when medications are brought and what steps are coming next).	The observer noted that the nurse told the patient, *“I’ll be back with your medications,”* without giving a timeframe or describing what would happen next. The observer encouraged the nurse to provide a more specific expectation, such as when the medications would arrive and what the patient should expect. This helps reduce uncertainty, particularly for patients who may be anxious or unfamiliar with the inpatient routine.
**Explaining Environment**	Explaining the hospital environment, workflow, and routine care processes (vital sign checks, medication passes, IV flushes, alarms, lab draws) as if the patient is entirely new.	The observer saw the staff member flushing an IV line without narrating the action or the expected sensation. *“Explain the environment… For instance, when flushing the line, [RN] could have explained what is happening or what they might feel (*e.g.*, temporary burning sensation). We do not want to assume the patient knows anything and feels prepared.”*
**Calm and Sincere**	Maintaining a calm and reassuring tone with the patient despite their uncertainty, pain, or concern.	The observer noted that the patient expressed discomfort and worry during the encounter, and the nurse immediately shifted into task-focused mode rather than acknowledging the patient’s feelings. The observer encouraged the nurse to maintain a calm, sincere presence by pausing briefly, offering reassurance, and validating the patient’s discomfort before proceeding with care—reinforcing that even short verbal reassurance (“*I hear you, and I’m here to help”*) can reduce anxiety and build trust. They also encouraged the staff to demonstrate calmness by remembering to “avoid rushing through tasks. Face the patient to maintain eye contact and use a calm tone. Confirm readiness before turning or repositioning.”
**Assume Nothing**	The hospital environment can be confusing and scary from the patient/family side. Assume nothing about the patient’s exposure and explain clearly all aspects of the task (including routine care steps like vitals, IV flushes, and medication passes, etc.) and what the patient will experience.	The observer noted that the staff member completed care tasks (including beginning a line flush) without describing what would happen next or what the patient should expect overnight. The observer coached the nurse by stating, *“Let’s work on explaining to the patient what to expect overnight, including vital checks, rounds, medication passes, lab draws, and noises in the hospital. Remember to assume that the patient has never been to the hospital.”*
**Personal Connection**	Deepen patient engagement through intentional listening, empathy, and the use of personal details shared by the patient to build rapport.	Observer noted staff member focused on the task at hand [and] could make better efforts in making a personal connection with talking to the patient and noting something important to her or could check on her lunch meal on the way. The patient and her sister in the room were very friendly and talkative.

## Data Availability

The data presented in this project are available on request from the corresponding author.

## Public Involvement Statement

Patients’ comments were used, and we spoke with patients about the quality improvement aspect of this project to include their perspective.

## Guidelines and Standards Statement

This manuscript was drafted against the SCOPE Guidelines for research.

## Use of Artificial Intelligence

This manuscript reflects original work and ideas developed entirely by the authors. In the review process, all reviewers noted that sentence structure in the paper could sometimes be simplified. One reviewer noted causality language and believed we overstated the novelty of our project. Therefore, to improve clarity and readability, we relied primarily on human peer review/editors and used limited support from Google and artificial intelligence tools to refine sentence structure and simplify overly complex phrasing, as well as to spot check for where we had causality language or overstated the novelty of our paper. AI provided alternative phrasing. These tools were used exclusively for language optimization—not for generating content, concepts, ideas, thoughts, or for re-arranging the paper’s structure, flow, or design—or used for anything other than simplifying complex sentences and tightening language.
